# Artificial Intelligence-Based Risk Stratification in Obesity Care: From Diagnosis to Personalised Treatment Pathways

**DOI:** 10.3390/diagnostics16101461

**Published:** 2026-05-11

**Authors:** Simona Wójcik, Monika Tomaszewska, Anna Rulkiewicz

**Affiliations:** 1 LUX MED, ul. Szturmowa 2, 02-678 Warsaw, Poland; 2 Wyższa Szkoła Nauk Medycznych LUX MED, ul. Szturmowa 2, 02-678 Warsaw, Poland

**Keywords:** obesity, artificial intelligence, machine learning, risk stratification, phenotyping, digital therapeutics, behavioural coaching, electronic health records, wearable devices, genomics, clinical decision support

## Abstract

**Background/Objectives:** Obesity is a chronic, relapsing disease with a widening gap between clinical need and the availability of specialist care. Artificial intelligence (AI) may enable earlier risk detection, more precise phenotyping, and scalable behavioural support across obesity treatment pathways. This narrative review synthesises contemporary AI applications across the obesity care continuum and evaluates their translational readiness. **Methods:** A targeted search of PubMed/MEDLINE and Google Scholar (January 2024–January 2026) was conducted, complemented by citation chaining. Evidence was synthesised across four domains: (1) risk prediction and screening, (2) environmental and behavioural determinants, (3) multimodal phenotyping and precision stratification, and (4) AI-enabled lifestyle interventions and behavioural coaching (AIBC). **Results**: Electronic health record (EHR)-based models demonstrate clinically useful discrimination for early risk identification. Multimodal approaches refine stratification beyond body mass index (BMI)-centric classification. AI-enabled behavioural coaching (AIBC) platforms show emerging evidence of clinically meaningful weight loss, including non-inferiority to human coaching; however, long-term effectiveness, generalisability, and equity remain insufficiently established. **Conclusions:** AI is positioned to become a core enabler of personalised obesity pathways. Safe translation requires external validation, bias auditing, transparent reporting, human oversight, and post-deployment surveillance aligned with clinical guidelines and regulatory expectations.

## 1. Introduction

### 1.1. Background and Rationale

Obesity has emerged as one of the defining health challenges of the 21st century. Recent global estimates suggest that approximately one in eight individuals is living with obesity, with prevalence rising across virtually all age groups and regions [1]. The economic burden is equally alarming: obesity and its related complications are estimated to account for healthcare and productivity costs comparable to several percent of global GDP. The impact is not evenly distributed. A growing majority of people with obesity now live in low- and middle-income countries, where rapid nutrition transition, urbanisation, and constrained health-system capacity intersect [1]. Against this backdrop, traditional models of obesity care—episodic visits, limited contact time, and fragmented services—are poorly suited to managing a complex, chronic, and relapsing condition that requires continuous monitoring, behavioural support, and timely adjustment of therapy.

At the same time, there is a profound mismatch between the scale of need and the availability of specialised obesity care. Multidisciplinary services capable of delivering evidence-based lifestyle interventions, pharmacotherapy, and metabolic and bariatric surgery remain scarce and unevenly distributed, even in high-income settings. Long waiting times, geographic barriers, and workforce shortages further limit access. The result is a persistent treatment gap: large numbers of individuals with obesity either receive no structured care at all or only intermittent, low-intensity interventions that are unlikely to produce durable weight loss or meaningful improvements in obesity-related complications. There is an urgent need for scalable, cost-effective approaches that can extend the reach of specialist expertise, personalise treatment intensity to individual risk and response, and provide continuity of support between in-person encounters.

Artificial intelligence (AI) has rapidly advanced from a research topic to a practical component of contemporary health care, offering precisely the kinds of capabilities that could help address these gaps. In diagnostic disciplines such as radiology, pathology, dermatology, and cardiology, machine learning and deep learning systems now routinely achieve performance comparable to human experts in image interpretation and pattern recognition [2,3]. Beyond diagnostics, AI is increasingly embedded in electronic health records and hospital information systems, where it supports tasks such as clinical coding, triage, risk prediction, and resource allocation [4]. Generative AI models, including large language models, have begun to transform clinical documentation workflows by drafting encounter notes, discharge summaries, and referral letters, reducing administrative burden and potentially alleviating clinician burnout. The breadth of these emerging capabilities—from medical note generation and patient triage to consultation support and preventive programme design—has been outlined in early appraisals of large language models in clinical settings, highlighting both the transformative potential and the persistent limitations of current systems, including the inability to replace direct physician–patient interaction [5]. Taken together, these advances reflect a qualitative shift in AI’s role in medicine: from experimental tool to embedded infrastructure. In obesity care specifically, where sustained behaviour change, early risk identification, and responsive treatment adaptation are central but chronically under-resourced, AI offers capabilities that are directly relevant—not merely aspirational. Integration of multimodal data from electronic records and wearable devices creates new opportunities for continuity of support that episodic clinic visits cannot provide [6].

### 1.2. Aim and Scope

This narrative review synthesises current applications of artificial intelligence across the obesity care continuum and evaluates their translational readiness for clinical practice. Evidence is organised across four complementary domains: early risk prediction, environmental and behavioural modelling, multimodal phenotyping, and AI-enabled behavioural coaching. Implementation and governance considerations are addressed in parallel. Detailed thematic scope and domain definitions are provided in Section 2.

### 1.3. Definitions and Functional Taxonomy of AI in Obesity Care

To reduce conceptual ambiguity and to maintain consistency across heterogeneous evidence, we organise AI applications by clinical function within obesity pathways (prediction, stratification, decision support, behavioural delivery, and generative interfaces). In obesity research and clinical practice, “artificial intelligence” refers to a heterogeneous set of computational approaches that differ in clinical purpose, data modalities, level of autonomy, and position within care pathways. To improve conceptual clarity, this review adopts a functional taxonomy that organises AI applications by what they do within obesity care. Firstly, predictive machine learning/deep learning (ML/DL) models aim to forecast risk and clinically relevant outcomes. In obesity, this includes prediction of incident obesity within predefined windows, risk of cardiometabolic complications, likelihood of response to lifestyle or pharmacological treatment, and longer-term weight trajectories or relapse. These models are typically trained on electronic health records, cohort data, and wearable-derived signals, and they serve as “upstream” components that support early detection and triage [2,4]. Secondly, AI supports risk stratification and multidimensional phenotyping beyond BMI-centric classification. Phenotyping models integrate multimodal data, including imaging-derived measures of visceral and ectopic adiposity, including magnetic resonance imaging (MRI), computed tomography (CT), dual-energy X-ray absorptiometry (DXA), metabolic biomarkers, and omics profiles (e.g., metabolomics, microbiome), and genetic information (single-nucleotide polymorphisms and polygenic risk scores). The goal is to delineate biologically and clinically meaningful subtypes of obesity that may differ in pathophysiology, prognosis, and treatment responsiveness [7,8]. Thirdly, clinical decision support systems (CDSS) and pathway-level orchestration tools embed predictive and phenotyping outputs into structured workflows. Examples include procedure selection support (pharmacotherapy versus metabolic and bariatric surgery), escalation or de-escalation of intervention intensity, monitoring strategies, and multidisciplinary care coordination. In this view, AI is not merely a model but part of an operational architecture that mediates how evidence-informed decisions are executed over time [9].

**Figure 1:**
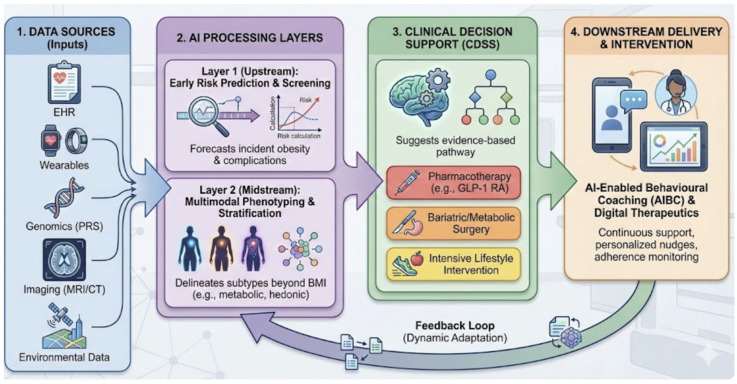
Functional architecture of AI-enabled obesity care pathways across four operational layers: (1) data inputs (EHR, wearables, genomics, imaging, and environmental data). (2) AI processing comprising upstream risk prediction and midstream multimodal phenotyping. (3) CDSS generating pathway recommendations across pharmacotherapy, bariatric/metabolic surgery, and lifestyle intervention. (4) Downstream delivery through AIBC and digital therapeutics, with a feedback loop linking monitoring outputs to upstream models. Abbreviations: EHR—electronic health record; PRS—polygenic risk score; MRI—magnetic resonance imaging; CT—computed tomography; GLP-1 RA—glucagon-like peptide-1 receptor agonist; AIBC—AI-enabled behavioural coaching; CDSS—clinical decision support system.

Fourthly, AI-enabled lifestyle interventions and AI-enabled behavioural coaching (AIBC) operationalize continuous, personalised behavioural support. AIBC systems may combine conversational agents, just-in-time adaptive interventions (JITAI), hybrid “digital twin” components, and engagement strategies such as gamification [10]. Their distinguishing feature is dynamic tailoring of content, frequency, and tone based on multimodal inputs from wearables, self-reports, and contextual signals, enabling a persistent “background layer” of support across treatment modalities [11,12]. Fifthly, generative AI, particularly large language models, functions as a human-facing interface between AI infrastructure and clinicians and patients. Typical use cases include documentation drafting, generation of patient education materials aligned with literacy and language needs, and interactive communication support. However, generative systems introduce distinct risks, notably hallucinations and guideline discordance, which necessitate explicit safeguards and human oversight [5,13]. Empirical evaluations of LLM performance on standardised medical examinations have demonstrated that these systems can achieve passing-level accuracy across clinical domains, yet exhibit systematic weaknesses in questions requiring nuanced clinical reasoning and image interpretation, underscoring the need for domain-specific validation before deployment [13]. Finally, from an implementation perspective, it is useful to distinguish clinician-facing tools (supporting professional decision-making) from patient-facing tools (delivering recommendations directly to patients), as these categories differ materially in acceptable risk thresholds, regulatory expectations and requirements for human-in-the-loop oversight [14,15]. This taxonomy provides the organising frame for the evidence synthesis that follows (Figure 1).

## 2. Methods

This manuscript is a narrative review. This format was selected to synthesise rapidly evolving and methodologically heterogeneous evidence on AI applications in obesity care, spanning predictive modelling, multidimensional phenotyping, AI-enabled behavioural coaching, and clinical governance. Given the breadth of data modalities (EHR, imaging, wearables, genomics, and digital therapeutics) and the variability in study designs, populations, and outcomes, a narrative approach was deemed the most appropriate to integrate findings across domains and to identify convergent themes, translational gaps, and implementation considerations.

A targeted literature search was conducted in PubMed/MEDLINE and Google Scholar. The search was deliberately restricted to publications from January 2024 to January 2026. This narrow temporal window was selected for three reasons. Firstly, the AI landscape in obesity care has undergone a qualitative shift in this period, marked by the first regulatory clearances of AI-driven digital therapeutics for weight management, the publication of the WHO guideline on GLP-1 RA [16] incorporating behavioural adjuncts, and the emergence of landmark RCTs comparing AI-enabled coaching with human-delivered programmes. Secondly, the breadth of AI applications in clinical settings—including documentation, triage, and patient communication—has been outlined in early appraisals of large language models in healthcare [5,13], while AI specifically in obesity has been addressed in recent comprehensive reviews [17,18]. Restricting our search window avoids redundant coverage while ensuring focus on the translational frontier. Thirdly, the rapid evolution of generative AI—including the clinical deployment of large language models for patient education and documentation—has introduced an entirely new category of tools that did not exist in earlier review periods. The search was last updated on 5 February 2026 to ensure the inclusion of the most recent evidence available at the time of manuscript preparation. We acknowledge that restricting the primary search to 2024–2026 may exclude foundational pre-2024 studies; older landmark evidence was therefore incorporated through systematic citation chaining from included reviews and consensus statements.

Key search terms were combined using Boolean operators and included “obesity” OR “adiposity” OR “bariatric” AND “artificial intelligence” OR “machine learning” OR “deep learning” OR “large language model” OR “generative AI” OR “digital therapeutic” OR “behavioural coaching” OR “JITAI” OR “wearables” OR “EHR” OR “electronic health record” OR “phenotyping” OR “polygenic risk score” OR “microbiome” OR “metabolomics” OR “MRI” OR “CT” OR “DXA”. Reference lists of highly relevant reviews and consensus statements were additionally screened to identify further eligible studies (snowballing).

The following exact search strings were applied across all databases. Search strategies were adapted to the syntax and indexing structure of each database. In PubMed/MEDLINE, the following query was used: (“Obesity”[MeSH] OR obes*[tiab] OR overweight[tiab] OR adiposity[tiab] OR “weight loss”[tiab] OR bariatric[tiab] OR “bariatric surgery”[tiab]) AND (“Artificial Intelligence”[MeSH] OR “Machine Learning”[MeSH] OR “machine learning”[tiab] OR “deep learning”[tiab] OR “large language model*”[tiab] OR “generative AI”[tiab] OR “digital therapeutics”[tiab] OR “behavioral coaching”[tiab] OR “behavioural coaching”[tiab] OR JITAI[tiab] OR wearable*[tiab] OR “electronic health record*”[tiab] OR phenotyp*[tiab] OR “polygenic risk score*”[tiab] OR microbiome[tiab] OR metabolomic*[tiab] OR MRI[tiab] OR CT[tiab] OR DXA[tiab]). In Embase, the following search was applied: (‘obesity’/exp OR obes*:ti,ab OR overweight:ti,ab OR adiposity:ti,ab OR ‘weight loss’:ti,ab OR bariatric:ti,ab OR ‘bariatric surgery’:ti,ab) AND (‘artificial intelligence’/exp OR ‘machine learning’/exp OR ‘machine learning’:ti,ab OR ‘deep learning’:ti,ab OR ‘large language model*’:ti,ab OR ‘generative AI’:ti,ab OR ‘digital therapeutics’:ti,ab OR ‘behavioral coaching’:ti,ab OR ‘behavioural coaching’:ti,ab OR JITAI:ti,ab OR wearable*:ti,ab OR ‘electronic health record*’:ti,ab OR phenotyp*:ti,ab OR ‘polygenic risk score*’:ti,ab OR microbiome:ti,ab OR metabolomic*:ti,ab OR MRI:ti,ab OR CT:ti,ab OR DXA:ti,ab). In the Cochrane Library, the search string was obesity AND (“artificial intelligence” OR “machine learning” OR “digital intervention” OR “digital therapeutics” OR “behavioral coaching” OR “behavioural coaching”). In Google Scholar, the following separate queries were entered: obesity AND (“artificial intelligence” OR “machine learning” OR “deep learning”), obesity AND (“digital intervention” OR “digital therapeutics” OR “behavioral coaching” OR “behavioural coaching”), and obesity AND (“large language model” OR “generative AI” OR wearable* OR phenotyping OR microbiome OR metabolomics).

We included (i) peer-reviewed review articles, consensus statements and position papers addressing AI in obesity, metabolic and bariatric surgery, or obesity-related cardiometabolic risk; (ii) original studies using ML/DL to predict obesity risk, treatment response or clinically relevant outcomes; (iii) studies on AI-enabled behavioural coaching or AI-driven digital lifestyle interventions, including randomised controlled trials when available; and (iv) studies integrating multimodal data for obesity phenotyping or risk stratification (e.g., genomics, imaging, metabolomics, microbiome). Included studies enrolled adult and/or paediatric populations with obesity, overweight, or at elevated risk of obesity.

We excluded (i) purely technical papers without clinical outcomes or clearly defined health-related endpoints; (ii) non-obesity populations, unless the primary aim was directly transferable to obesity management (e.g., diabetes prevention programmes with a substantial overweight/obesity subgroup); (iii) editorials without substantive synthesis; and (iv) non-English publications.

Titles and abstracts were screened for relevance, followed by full-text assessment of key articles. Evidence was synthesised thematically rather than quantitatively, with studies grouped into four domains: (1) early risk prediction and screening (EHR-based models and cohort analyses); (2) environmental and behavioural determinants (including image-based built-environment models); (3) multidimensional phenotyping and precision risk stratification (genomic and multimodal models); and (4) AI-enabled behavioural coaching and digital therapeutics, including implications for clinical governance. Priority was given to high-quality evidence (systematic reviews, large cohorts, and randomised trials) and to clinically actionable models with clear endpoints and validation approaches. Given the narrative review design, no formal risk-of-bias tool was applied across all included studies. To mitigate selection bias and enhance interpretability, studies were prioritised on the basis of (1) sample size and cohort representativeness, (2) external validation or multi-site design, (3) clinically meaningful primary endpoints, and (4) transparent reporting of model performance metrics.

The authors acknowledge the use of an image generation model (Gemini 2.5 Flash Image/Nano Banana) to facilitate the conceptualization and initial drafting of the schematic illustrations presented in this review. All AI-assisted visual elements were subsequently critically reviewed, refined, and validated by the authors to ensure scientific accuracy and alignment with the manuscript’s content.

In the sections that follow, we first summarise upstream AI for risk prediction and screening, then review environmental and behavioural modelling, and subsequently discuss multimodal phenotyping and precision stratification. We then evaluate downstream delivery tools, including AIBC and digital therapeutics, and conclude with implementation requirements for safety, equity, governance, and clinical integration. The functional taxonomy introduced in Section 1.3 provides the definitional frame for the evidence synthesis.

## 3. Evidence Synthesis AI Applications Across the Obesity Care Continuum

**Figure 2:**
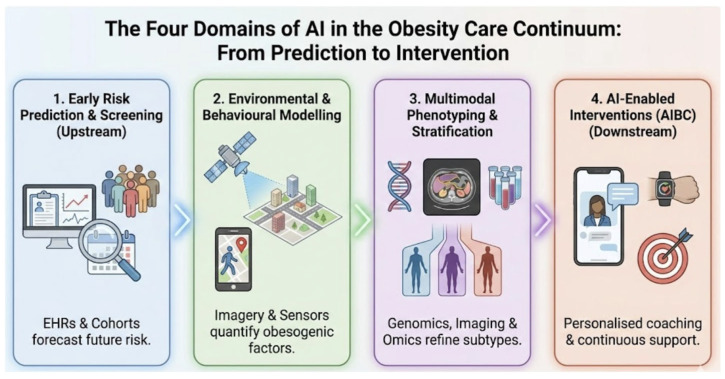
The four domains of AI applications across the obesity care continuum: from prediction to intervention. Domain 1 (Early Risk Prediction and Screening, upstream): EHR-based and cohort-derived models forecast incident obesity and cardiometabolic complications. Domain 2 (Environmental and Behavioural Modelling): Satellite and street-level imagery analysed by CNNs quantifies obesogenic environmental exposures at neighbourhood scale. Domain 3 (Multimodal Phenotyping and Stratification): Integration of genomics, imaging, and multi-omics data delineates biologically meaningful obesity subtypes beyond BMI. Domain 4 (AI-Enabled Interventions, AIBC, downstream): Personalised coaching platforms deliver continuous, adaptive behavioural support. Abbreviations: EHR—electronic health record; CNN—convolutional neural network; BMI—body mass index; AIBC—AI-enabled behavioural coaching.

The following synthesis organises current evidence into four clinically grounded domains. For each domain, we assess translational readiness with reference to validation design, endpoint selection, workflow fit, and governance requirements. The structure moves from upstream prediction to downstream delivery, reflecting the sequence in which AI tools encounter patients across an obesity care pathway, as illustrated in Figure 2.

### 3.1. Domain 1: Early Risk Prediction and Screening (EHR, Cohorts, Wearables as Inputs)

A central translational question is whether AI models can identify high-risk trajectories early enough to enable preventive action before complications emerge. A recent narrative review by Azmi et al. (2025) [17] provides an upstream anchor by synthesising three pillars of ML and deep learning applications in obesity: EHR-based prediction of incident obesity, image-based quantification of built environment correlates using satellite and street-level data, and multimodal models integrating genetic and phenotypic variables for refined risk stratification and phenotyping. In the taxonomy adopted here, these pillars primarily populate the screening and stratification layers of the pathway and can subsequently be operationalized through clinical decision support and AI-enabled behavioural coaching platforms that tailor intervention intensity and modality over time [17,18].

In relation to early screening, multiple studies have used electronic health records to predict incident obesity in children, including cohorts with up to approximately 11 million paediatric encounters. Algorithms such as random forests, gradient boosting, and deep neural networks are trained on longitudinal anthropometric trajectories, perinatal factors, comorbid diagnoses, medication exposures, and sociodemographic variables to identify children at high risk of developing obesity within prespecified prediction windows (for example, 1 to 5 years) [17,19,20]. These models are typically evaluated using hold-out test sets or cross-validation and achieve clinically useful discrimination across diverse health system datasets, supporting their potential use as screening tools to trigger targeted early life preventive interventions and more intensive follow-up for those most likely to exceed BMI thresholds in later childhood [19].

Evidence from adult cohorts further supports the feasibility of AI-based risk prediction. Within the Korean KNHANES programme, encompassing data from 21,100 adults, researchers tested multiple models to predict obesity risk [21]. Tree-based methods such as random forest and XGBoost analysed metabolic variables, including triglycerides, liver enzymes, and uric acid, with predictive performance varying by sex and age and the highest accuracy observed in younger adults [22]. Reported feature importance profiles suggested differences across subgroups, for example, ALT ranking highly in men and triglycerides in women, indicating the potential value of stratified model development for personalised risk assessment [22].

Interpretability tools are a practical requirement for clinical adoption, not merely a technical refinement. In NHANES-derived models combining metabolic and behavioural variables, SHAP (SHapley Additive exPlanations) analysis—a game-theoretic approach that assigns each input variable a contribution value for individual predictions, enabling patient-level model interpretation—has identified daily step count, sedentary time, and fasting glucose as the strongest predictors of obesity risk—findings that translate directly into target-setting for lifestyle counselling. The ability to generate patient-level explanations alongside risk estimates substantially reduces the barrier to integrating ML outputs into consultation workflows [23]. One advantage of predictive models is their ability to capture nonlinear interactions that may be overlooked in standard BMI-based assessments—an important consideration given that obesity pathophysiology extends well beyond simple energy balance [24]. For example, rising glucose in combination with low physical activity and prolonged sedentary exposure in early childhood may increase the probability of subsequent obesity over multi-year horizons, suggesting a potential role for AI-enabled screening integrated into electronic health records to identify children requiring intensified preventive measures, including behavioural and family-based support. Large-scale evidence synthesis further supports the temporal predictive capacity of ML models in obesity. Kalhori et al. conducted a systematic review including 10 studies across 411,000 participants with follow-up of one to five years, selected from an initial pool exceeding 6000 publications. Inputs included age, sex, lifestyle variables, blood biomarkers, and anthropometric measures, enabling machine learning algorithms to model nonlinear relationships and interacting factors that can be difficult to capture with traditional statistical approaches [20].

**Strength of evidence and remaining gaps.** The evidence base for EHR-based obesity risk prediction is the most mature among the four domains, supported by multiple large-cohort retrospective studies (*n* up to 11 million encounters), systematic review-level synthesis, and consistent demonstration of clinically useful discrimination (AUC 0.80–0.93 across age subgroups). Interpretability methods such as SHAP have been applied to generate patient-level explanations, strengthening the case for clinical adoption. However, critical gaps persist. Almost all models rely on internal validation (hold-out or cross-validation) within the originating health system, and external validation across geographically or ethnically distinct populations remains rare. No prospective implementation study has yet demonstrated that embedding these models into clinical workflows reduces obesity incidence or improves downstream health outcomes. Additionally, the predominance of paediatric cohorts from the United States limits generalisability to adult populations and non-US health systems. Future work should prioritise prospective, multi-site validation with pre-registered clinical endpoints and equity-stratified performance reporting.

### 3.2. Domain 2: Environmental and Behavioural Determinants (Built Environment, Imagery)

The built environment is an established determinant of obesity risk, with systematic evidence demonstrating that environmental components—including access to recreational facilities, food environments, and neighbourhood walkability—are integral to effective prevention programmes [25]. Convolutional neural networks (CNNs) have been applied to satellite and street-level imagery to quantify such features at neighbourhood or census-tract level at scale [17]. In these studies, CNNs are trained on high-resolution images from platforms such as Google Street View or satellite providers to extract latent visual features related to land-use mix, building density, road structure, availability of green space, sidewalks, and recreational facilities. These image-derived representations are then linked to population-level BMI or obesity prevalence data from surveys or health records, enabling identification of environmental configurations that correlate with higher or lower obesity burden. This approach supports the scalable, standardised assessment of obesogenic and protective environments across large geographic areas, complementing labour-intensive environmental audits and informing urban planning and public health policy.

Recent advances have substantially scaled, refined, and integrated these CNN-based methods with advanced geospatial analytics, demonstrating their maturity as actionable public health tools [17]. Chen et al. (2025) [26] conducted a study across 10 major US metropolitan areas (covering >1000 census tracts), employing deep CNN architectures to extract built environment features from both high-resolution satellite imagery and Google Street View panoramas, achieving 20–30% explained variance in neighbourhood-level adult obesity prevalence—significantly outperforming traditional socioeconomic status (SES) indicators such as income, education, and deprivation indices. The models identified specific obesogenic configurations (e.g., high building density with low walkability) and enabled geospatial hotspot mapping via clustering algorithms, offering urban planners prioritised intervention zones with estimated impact on obesity reduction [26].

Similarly, a 2024 Missouri statewide geospatial analysis (published early 2025) integrated ResNet-50 CNN-extracted visual features from 10 m-resolution satellite imagery with spatial econometric models (spatial lag/error specifications) and Local Indicators of Spatial Association (LISA), predicting obesity prevalence with high accuracy across urban, suburban, and rural census tracts. Key modifiable drivers included low vegetation density, single-lane road dominance, and fragmented green space, with rural areas showing distinct patterns of geographic isolation amplifying risk. The spatial models outperformed non-spatial baselines (R^2^ improvement of 15–25%), highlighting geographic autocorrelation as a critical confounder absent in traditional audits [27]. A 2025 multi-city *Scientific Reports* study spanning 19 diverse US urban areas (varying climate zones and demographics) further validated the approach using multi-modal CNN ensembles on Street View and Landsat satellite data, explaining ~25% of variance in both obesity prevalence and self-reported physical activity. Walkable streetscapes with ≥30% green coverage emerged as consistently protective (r = −0.35 to −0.45), while car-centric designs (wide roads, low mixed-use) correlated with higher BMI trajectories. The study demonstrated cross-city generalizability through transfer learning, reducing retraining needs for policy scalability [28].

Across these studies, CNN-based imagery analysis has demonstrated a consistent ability to identify specific, modifiable environmental configurations—low walkability, sparse green space, car-centric road design—that predict neighbourhood-level obesity prevalence at scale and with accuracy that manual audits cannot match. The practical implication is that area-level environmental risk scores derived from satellite and street-view data could serve as inputs for targeted public health resource allocation, informing where AIBC programmes or urban redesign investments are likely to have the greatest preventive impact. Rural–urban differences in risk patterns, as demonstrated in the Missouri analysis, suggest that these tools should be deployed with geographically stratified models rather than universal algorithms [28].

**Strength of evidence and remaining gaps.** Domain 2 evidence is anchored by several large-scale geospatial studies employing robust CNN architectures across diverse US metropolitan areas, achieving 20–30% explained variance in neighbourhood-level obesity prevalence and outperforming conventional SES indicators. The use of spatial econometric models and transfer learning across cities further strengthens methodological rigour. Nevertheless, these models operate exclusively at the population level; no study has yet linked area-level environmental risk scores to individual-level clinical decision-making or intervention outcomes. Geographic coverage remains confined to the United States, leaving it unclear whether identified obesogenic configurations (e.g., low walkability, sparse green space) replicate in European, Asian, or low- and middle-income settings where urbanisation patterns differ. Prospective studies evaluating whether CNN-derived environmental vulnerability indices can inform resource allocation decisions—and whether acting on such information reduces obesity prevalence—are needed.

### 3.3. Domain 3: Multimodal Phenotyping and Precision Risk Stratification (Genomics, Imaging, Omics)

**Figure 3:**
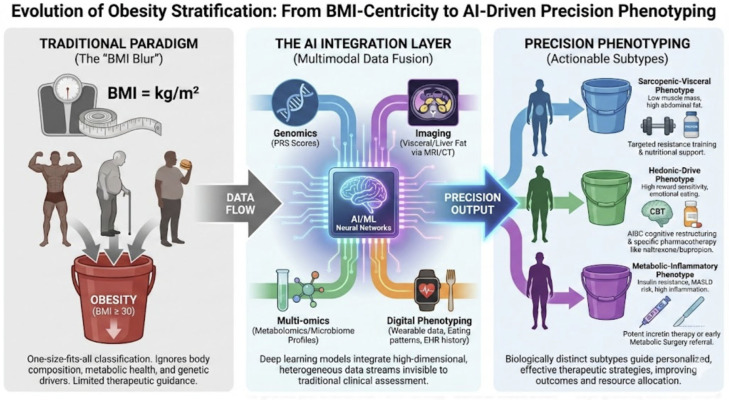
Evolution of obesity stratification: from BMI-centricity to AI-driven precision phenotyping. The left panel illustrates traditional BMI-based classification, which applies a single anthropometric threshold irrespective of body composition, metabolic health, or genetic background. The central panel depicts the AI integration layer, where deep learning models fuse multi-omics profiles (genomics, metabolomics, microbiome), imaging-derived measures of visceral adiposity (MRI/CT), and digital phenotyping data from wearables and EHR to generate high-dimensional feature representations. The right panel shows the resulting precision phenotypes—including sarcopenic-visceral, hedonic-drive, and metabolic-inflammatory subtypes—each associated with distinct pathophysiology and proposed targeted therapeutic strategies ranging from resistance training and nutritional support to GLP-1-based pharmacotherapy and cognitive-behavioural interventions. Abbreviations: BMI—body mass index; MRI—magnetic resonance imaging; CT—computed tomography; GLP-1—glucagon-like peptide-1; CBT—cognitive behavioural therapy; MASLD—metabolic dysfunction-associated steatotic liver disease; AIBC—AI-enabled behavioural coaching.

Multimodal ML and deep learning models integrate genetic information, including single-nucleotide polymorphisms and polygenic risk scores, with clinical, behavioural, and environmental covariates to refine individual risk estimation and delineate clinically meaningful obesity phenotypes. Methods such as regularised regression, tree-based ensembles, and neural networks combine hundreds to thousands of genetic variants with phenotypic data, such as BMI trajectories, metabolic markers, and lifestyle factors [17] (Figure 3).

Across the studies reviewed, joint models generally outperform those using either genomic or clinical data alone for predicting obesity status or related cardiometabolic outcomes. Unsupervised clustering on integrated feature sets has also been used to identify subgroups with differing pathophysiological profiles and potentially differential response to interventions, providing a methodological basis for more personalised prevention and management strategies [7,17,29]. This advantage has been quantified systematically by Kim et al. (2025), who reported that multimodal AI models outperformed their unimodal counterparts in 91% of evaluated studies, with performance gains ranging from 6 to 33% depending on the combination of data modalities and clinical endpoint [8]. Complementary evidence from Ruan et al. (2025) [30] demonstrates that EHR data alone—including comorbidity trajectories, medication sequences, and longitudinal anthropometric patterns—can support deep phenotyping of obesity subtypes without requiring omics or imaging inputs, suggesting a lower-cost pathway to clinically actionable stratification in settings where multimodal data are unavailable [30].

Recent multimodal modelling efforts have further validated and extended this paradigm by demonstrating superior predictive performance and emerging clinical utility for treatment response stratification. A 2026 study by Xiao et al. (2026) [31] developed an ML-derived polygenic risk score (PRS) from UK Biobank data (n = 482,700 individuals), integrating hundreds of thousands of SNPs with longitudinal phenotypic, lifestyle, and environmental covariates to forecast BMI trajectories across the life course; the joint model outperformed standard PRS by 12–18% in AUC for obesity incidence and persistence, capturing non-linear gene–environment interactions missed by univariate approaches. Similarly, a 2025 scoping review by Vahid et al. (2026) [29] synthesised over 100 studies using regularised regression, tree-based ensembles, and neural networks to combine genetic variants with metabolomic, microbiome, and imaging data (e.g., visceral fat distribution from MRI/CT), consistently showing multimodal models superior to genomic or clinical silos (mean AUC gain 0.08–0.15) for cardiometabolic outcomes [29,31].

Unsupervised clustering on these integrated datasets has yielded actionable phenotypes with differential intervention responses. Emerging research identifies metabolically distinct obesity subtypes with differential pharmacotherapy response profiles. Phenotype-guided pharmacotherapy matching represents an active research direction requiring prospective validation. Jia et al. (2025) [7] applied DDRTree unsupervised clustering to routine clinical data from 18,733 participants, identifying five metabolic phenotypes with distinct cardiovascular and type 2 diabetes risk profiles and proposed phenotype-specific treatment strategies, including GLP-1 prioritisation for subtypes characterised by high visceral adiposity [7]. These advances provide a methodological foundation for precision obesity care, transforming abstract risk scores into pathway-specific decision aids that inform whether patients require lifestyle intensification, early pharmacotherapy, or MBS referral [7].

**Strength of evidence and remaining gaps.** Multimodal phenotyping models consistently demonstrate superior predictive performance over unimodal approaches—quantified at 6–33% improvement in a recent comprehensive review [8]—and unsupervised clustering has identified clinically meaningful subtypes with differential cardiometabolic risk profiles. The integration of ML-derived polygenic risk scores with lifestyle and environmental data in UK Biobank (n = 482,700) represents the largest-scale validation to date. However, this domain has the widest gap between methodological promise and clinical translation. No prospective trial has tested whether phenotype-guided treatment allocation improves outcomes compared with standard care. Most models are trained on data from European-ancestry biobanks, raising equity concerns for underrepresented populations. The high cost and limited availability of multi-omics and advanced imaging data restrict scalability, although EHR-based deep phenotyping approaches offer a lower-cost alternative. Validation against clinically actionable endpoints—such as differential response to GLP-1 RA versus cognitive-behavioural therapy by phenotype—should be a priority for the next generation of studies. A recent comprehensive review spanning mechanistic, clinical, and population-level AI applications in obesity further underscores the need for federated learning and privacy-preserving approaches to enable multi-site model development without centralising sensitive patient data [32].

### 3.4. Domain 4: AI Enabled Lifestyle Interventions and Behavioural Coaching (AIBC)

Building on upstream prediction and stratification, Hallock et al. (2026) extend the role of AI in obesity medicine from identifying who is at risk to dynamically tailoring how care is delivered over time [6]. Whereas the *Diagnostics* review focuses on models trained on EHR, environmental imagery and genomic data to quantify and explain obesity risk, Hallock and colleagues describe how similar multimodal data streams can be integrated into AI enabled behavioural coaching platforms and clinical decision support systems that personalise intervention intensity, content and mode of delivery [17]. In this framework, outputs from predictive models, such as paediatric risk scores, environmental vulnerability indices or genetically informed phenotypes, serve as inputs for tools that adjust lifestyle coaching, inform choices across lifestyle modification, pharmacotherapy and metabolic or bariatric surgery, and support continuous monitoring and adaptation of treatment.

AI-enabled behavioural coaching refers to digital interventions that use AI to deliver continuous, personalised lifestyle support for individuals with obesity. Systems may combine conversational agents, just-in-time adaptive interventions, digital twin components, and engagement mechanisms such as gamification [11]. Personalization is typically driven by multimodal data streams, including step counts, heart rate, and sleep metrics from wearables, alongside self-reported diet, mood, and contextual factors. On this basis, platforms tailor the timing and frequency of prompts, the content and tone of messages, and the specific tasks proposed to the user, aiming to sustain engagement and adherence over time [12,33].

Emerging clinical evidence suggests that AIBC interventions can achieve non inferior outcomes compared with standard care and may be associated with clinically meaningful weight loss, often defined as at least 5 percent of baseline body weight, alongside improvements in metabolic parameters including glycaemic control, lipid profiles, and blood pressure. However, the evidence base remains constrained by small samples, short follow up, and heterogeneity in intervention design, target populations, and outcome definitions, limiting generalizability and precluding firm conclusions about long term effectiveness or cost effectiveness [34]. A 2025 systematic review and meta-analysis of smartphone-based nutritional interventions confirmed modest but statistically significant effects on weight loss and body composition in adults with overweight or obesity, while underscoring that app-only interventions without additional AI personalisation or human support yield smaller effect sizes than hybrid approaches [35]. A further systematic review and meta-analysis demonstrated significant reductions in body weight, BMI, and waist circumference with nursing-led telemedicine interventions [36], reinforcing the effectiveness of remotely delivered behavioural support as a scalable foundation for AIBC platforms. In addition, many AIBC solutions have been developed independently of formal clinical practice guidelines or clear regulatory frameworks for digital therapeutics, raising questions about guideline concordance, data governance and accountability. Randomised evidence has begun to address these translational requirements [37]. In a trial embedded in the Diabetes Prevention Programme framework, an AI-powered lifestyle intervention achieved non inferior weight loss and glycaemic outcomes compared with standard human delivered coaching among adults at high risk of type 2 diabetes, many of whom had overweight or obesity [37]. The AI arm delivered automated, algorithmically tailored content and feedback, while the control arm followed the conventional DPP model with trained human coaches. Comparable outcomes support the feasibility of scaling behavioural programmes through AI while maintaining clinically meaningful effects [6].

**Strength of evidence and remaining gaps.** Domain 4 has produced the strongest direct evidence of patient benefit, including a landmark RCT demonstrating non-inferiority of AI-delivered coaching to human coaches within the Diabetes Prevention Programme framework [37]. Additional mixed-methods and feasibility studies support the acceptability and short-term effectiveness of AI-assisted dietary and physical activity interventions. However, the evidence base remains thin in several respects: sample sizes are modest, follow-up rarely exceeds 12 months, and outcome definitions vary across studies, precluding quantitative synthesis. Cost-effectiveness data are absent. Critically, most AIBC platforms have been evaluated in high-income, digitally literate populations, leaving generalisability to low-resource settings undemonstrated. The extension of AI-driven coaching to clinically sensitive populations such as pregnant women remains at the protocol stage. Regulatory pathways for digital therapeutics in obesity are emerging (e.g., FDA clearance of Signos) but are not yet standardised across jurisdictions. Long-term, multi-site RCTs with equity-stratified endpoints and health-economic evaluation are the most pressing evidence needs.

### 3.5. Cross Cutting Synthesis: Translational Readiness, Guidelines, Regulatory Milestones, and Deployment Signals

The convergence of guideline endorsement, regulatory clearance, and commercial scaling that occurred between 2024 and 2026 represents a qualitative inflexion point for AI in obesity medicine—one that moves the field beyond proof-of-concept and into questions of implementation fidelity, equity of access, and post-deployment governance. In late 2025, the World Health Organization issued its first global guideline on GLP-1 receptor agonists for obesity treatment, providing conditional recommendations for long-term pharmacotherapy in adults alongside intensive behavioural interventions as essential adjuncts. This landmark document explicitly recognises the need for comprehensive, multidisciplinary care integrating diet, physical activity, and professional support, directly validating the role of AI-enabled behavioural coaching (AIBC) as a scalable delivery mechanism for the behavioural pillar within personalised pathways combining lifestyle modification and pharmacotherapy [16]. Similarly, the 2025 Obesity Algorithm update from the Obesity Medicine Association incorporates AI-driven prediction and monitoring tools into its decision trees for risk assessment, phenotyping, and therapy selection across lifestyle, pharmacotherapy, and metabolic/bariatric surgery (MBS) pathways [38]. Concurrently, the Gastroenterology consensus algorithm for obesity evaluation leverages the 2025 Lancet diagnostic criteria (clinical obesity plus individual risk factors), creating visual pathways where AI phenotyping and risk stratification tools fit seamlessly as enablers of escalated, personalised care [6,39]. Regulatory milestones further underscore clinical viability. In 2025, the FDA cleared Signos as the world’s first AI-driven app paired with continuous glucose monitoring (CGM) for weight management, employing biomarker-informed nudges to optimise food and lifestyle choices based on real-time glucose responses [40]. These approvals establish a viable FDA pathway for AI obesity DTx, demonstrating regulatory feasibility while highlighting equity challenges (access/cost barriers) central to our ethical discussion.

**Figure 4:**
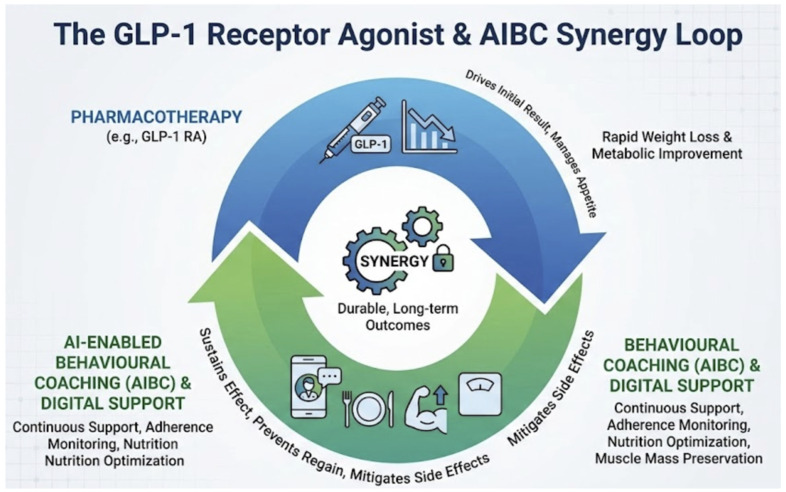
The GLP-1 receptor agonist and AI-enabled behavioural coaching (AIBC) synergy loop. GLP-1 pharmacotherapy drives rapid initial weight loss and metabolic improvement; AIBC provides continuous behavioural support, adherence monitoring, and muscle mass preservation, thereby mitigating common adverse effects and addressing the risk of weight regain following GLP-1 discontinuation. The synergistic interaction between pharmacological and digital therapeutic components is proposed as the dominant model for personalised obesity management in 2025–2026. Abbreviations: GLP-1 RA—glucagon-like peptide-1 receptor agonist; AIBC—AI-enabled behavioural coaching.

At the commercial level, the integration of GLP-1 pharmacotherapy with continuous AI-enabled monitoring is moving from concept to product. Allurion Technologies’ AllurionMeds platform—which pairs GLP-1 prescribing with AI-driven coaching and body composition tracking—exemplifies this model, targeting the clinically important problem of weight regain following GLP-1 discontinuation. Whether such integrated programmes produce durable outcomes beyond the pharmacotherapy phase remains an open question requiring prospective data (Figure 4) [41]. IQVIA’s analysis notes the rise in FDA-cleared over-the-counter CGMs (Abbott Lingo, Dexcom Stelo) integrated with AI apps for glucose-guided obesity management, addressing adherence gaps in GLP-1 therapy. Reports like Obesity Management: Advances in 2025 confirm AI + GLP-1s as dominant 2025 innovations, with market growth in CGM/AI platforms [42]. The pace of this transition from research to practice is itself a governance risk: when deployment outstrips validation, inequities in access and algorithmic harm can become entrenched before surveillance mechanisms are in place. The evidence reviewed here suggests that clinical maturity is uneven across domains—strongest in EHR-based prediction and RCT-tested AIBC, thinner in multimodal phenotyping and pathway orchestration—and that translation decisions should be calibrated accordingly. A comprehensive summary of the key studies reviewed across all four domains—including study design, population, sample size, data modality, AI method, primary outcome, explainability approach, validation status, and translational readiness level—is provided in Supplementary Table S1.

## 4. Discussion

This narrative review demonstrates that AI applications in obesity care have moved beyond proof-of-concept, yet the four domains examined differ markedly in translational maturity—the most robust evidence to date in EHR-based screening (Domain 1) and RCT-tested behavioural coaching (Domain 4), pending prospective multi-site validation, through scalable but population-level environmental modelling (Domain 2), to scientifically compelling but clinically unvalidated multimodal phenotyping (Domain 3). The domain-specific evidence profiles, including their respective strengths and remaining gaps, have been detailed in the preceding synthesis (Section 3.1, Section 3.2, Section 3.3 and Section 3.4 and Supplementary Table S1). The discussion that follows therefore focuses on contextualising these findings against prior reviews, extracting clinical implications per domain, and addressing governance requirements for safe translation.

These findings extend and complement several recent reviews. Azmi et al. (2025) provided a comprehensive catalogue of AI techniques applied to obesity but focused primarily on methodological description rather than translational readiness or governance requirements [17]. Huang et al. (2025) offered a broader perspective on AI in obesity risk prediction and management but did not systematically evaluate the AIBC landscape, regulatory milestones, or implementation barriers [18]. The present review adds three contributions that were not addressed by these earlier works: first, a functional taxonomy that explicitly maps AI tools to their position and role within clinical obesity pathways, from upstream screening to downstream behavioural delivery; second, a domain-by-domain assessment of translational readiness, including validation design and endpoint maturity; and third, the integration of rapidly emerging evidence on AI–GLP-1 pharmacotherapy synergies, FDA regulatory clearances, and WHO guideline endorsements that post-date the search windows of prior reviews. Importantly, the maturity gap between domains has practical implications for implementation sequencing. EHR-based screening tools and validated AIBC platforms are closest to deployment readiness and may yield the most immediate clinical returns, whereas multimodal phenotyping tools—though scientifically compelling—will require prospective validation in phenotype-guided treatment allocation trials before they can be recommended for routine use. This staged approach to adoption would allow health systems to accrue implementation experience and build governance infrastructure incrementally, rather than attempting simultaneous deployment across all AI modalities.

The majority of studies reviewed across all four domains are retrospective, single-centre, or single-health-system in design, and none of the EHR-based predictive models reviewed have undergone prospective, pre-registered implementation studies. Specific bias risks include selection bias from non-representative training cohorts, predominantly drawn from US and East Asian health systems with limited representation of ethnic minorities, lower-income populations, and non-English-speaking communities; label bias in EHR-based models arising from differential coding practices across institutions; and confounding by indication in AIBC studies, where participants who engage more consistently with digital platforms may systematically differ from those who do not. Notably, fewer than 20% of reviewed studies reported equity-stratified performance metrics across demographic subgroups. These limitations must be considered when interpreting reported discrimination statistics and when designing implementation studies.

A critical distinction exists between model performance in controlled research settings and expected performance in deployment conditions, where distribution shift, data quality degradation, and patient population heterogeneity routinely erode discrimination metrics. This gap is particularly relevant for obesity AI tools, which will be deployed across diverse health systems with varying coding practices, device uptake, and population demographics. The absence of post-deployment surveillance data for any of the AI tools reviewed—including FDA-cleared devices such as Signos—represents a significant gap. No published post-market performance data from real-world deployment of commercial AI obesity tools are available in the peer-reviewed literature at the time of writing. We call for mandatory post-deployment monitoring frameworks as a condition of clinical adoption, including pre-specified performance thresholds and demographic subgroup reporting.

From a clinical standpoint, several domain-specific implications deserve emphasis. In Domain 1, the consistent ability of EHR-based models to identify children and adults on high-risk obesity trajectories 1–5 years in advance suggests a concrete use case: embedding validated risk scores into routine well-child visits and annual health checks to trigger referral to preventive programmes, analogous to cardiovascular risk calculators already in clinical use. The sex- and age-specific feature importance profiles observed in the KNHANES and NHANES analyses further suggest that a single universal model may be suboptimal; instead, stratified models calibrated to local demographic profiles are likely to yield better positive predictive values and reduce alert fatigue in primary care settings. In Domain 2, the demonstration that CNN-derived environmental risk scores outperform conventional socioeconomic indicators for predicting neighbourhood-level obesity prevalence creates an opportunity for collaboration between public health agencies and urban planning authorities. Area-level environmental vulnerability indices could inform where to invest in walkability infrastructure, green space expansion, and AIBC programme deployment—linking AI-derived population intelligence to actionable policy levers.

In Domain 3, the identification of metabolically distinct obesity subtypes—such as the five phenotypes reported by Jia et al. [4] with differential cardiovascular and type 2 diabetes risk—raises the possibility of phenotype-guided treatment selection. If prospective trials confirm that, for example, patients with high visceral adiposity and insulin resistance benefit preferentially from early GLP-1 RA initiation while those with a hedonic-drive phenotype respond better to cognitive-behavioural approaches combined with naltrexone/bupropion, the clinical impact would be substantial. However, until such trials are completed, phenotype-guided pathways should be considered hypothesis-generating rather than ready for clinical adoption. Notably, Xiao et al. (2026) demonstrated that the prognostic impact of a high ML-derived polygenic risk score was substantially attenuated by adherence to a healthy lifestyle, suggesting that AI-based genetic risk stratification should be framed as motivational rather than deterministic—reinforcing the link between upstream phenotyping (Domain 3) and downstream behavioural coaching (Domain 4) [31].

In Domain 4, the non-inferiority of AI-delivered coaching to human coaches in the DPP trial carries direct implications for scalability and reimbursement. If replicated in larger, longer-duration trials with diverse populations, these findings would support the inclusion of AI-based behavioural interventions in national coverage frameworks, potentially expanding access to evidence-based obesity care in underserved areas where trained human coaches are scarce. The emerging model of combining GLP-1 pharmacotherapy with continuous AI-enabled behavioural monitoring—as exemplified by the Signos FDA clearance and the Allurion platform—represents a paradigm in which pharmacological and digital therapeutic modalities are co-prescribed rather than offered sequentially, though prospective evidence of additive benefit remains limited.

The clinical promise of AI in obesity care is inseparable from a set of governance challenges that deserve systematic attention. Chief among these are the risk of hallucination in generative systems deployed for patient education, the potential for algorithmic bias to amplify existing inequities in access to care, and the absence of clear accountability structures for patient-facing AI recommendations [6,18,43]. At the same time, many models are trained predominantly on data from high-income settings and majority populations, with limited representation of ethnic minorities and people living in low- and middle-income countries; this uneven data landscape increases the likelihood that predictions of risk, treatment response or adherence will be less accurate—and potentially harmful—for precisely those groups already facing structural barriers to care [6,44,45,46]. In response to such concerns, the World Health Organization has articulated six core principles for ethical AI in health—respect for autonomy, promotion of human well-being and safety, transparency and explainability, responsibility and accountability, inclusiveness and equity, and sustainability—while professional bodies such as the AMA and AHA emphasise model explainability, continuous monitoring for harm, and the preservation of clinician accountability [47]. However, current AIBC interventions in obesity rarely operationalize these frameworks in a systematic way, revealing a substantial gap between high-level guidance and day-to-day practice. Cultural and linguistic contexts must also be considered during implementation, as AI tools developed in high-income settings may embed assumptions that are poorly suited to the populations most affected by rising obesity prevalence. Translating these principles into practice requires attention to five specific requirements: prospective bias auditing across demographic subgroups before deployment; external validation in the local patient population, not only in the training cohort; defined human oversight protocols for all patient-facing outputs; explicit uncertainty communication to clinicians and patients; and post-deployment surveillance capable of detecting degradation in performance or new inequities as patient populations shift.

The extension of AI-enabled lifestyle interventions into clinically sensitive populations further amplifies the need for robust ethical and regulatory frameworks. A recent study of an AI-driven therapeutic lifestyle change (AI-TLC) programme in pregnant women with obesity illustrates this trajectory, using algorithmically tailored coaching to influence diet, physical activity, and other health behaviours during gestation. While such interventions hold promise for improving maternal metabolic status and potentially reducing adverse pregnancy outcomes, they also operate in a context where the stakes are uniquely high: any unintended effects of algorithmic misclassification, inappropriate advice, or unrecognised bias may impact not only the pregnant individual but also foetal development and long-term offspring health. When viewed alongside broader proposals for AI-enabled behavioural coaching and precision phenotyping in obesity, these findings suggest that regulatory expectations for validation, safety monitoring, transparency, and human oversight should be at least as stringent for AI systems targeting high-risk groups—such as pregnant women—as for conventional pharmacological or surgical interventions, including clear standards for informed consent, data governance, and equitable access [6,48]. Parity between AI coaching and human-delivered DPP has direct implications for reimbursement: digital therapeutics that demonstrate non-inferiority in RCTs should be evaluated under frameworks analogous to those applied to pharmacological or procedural interventions, including requirements for post-marketing surveillance and equity monitoring [37].

Algorithmic bias represents a structural risk that deserves explicit attention in the context of obesity AI tools. Models trained predominantly on high-income, majority-ethnicity populations risk amplifying rather than reducing health disparities—precisely for those groups most affected by rising obesity prevalence. Otokiti et al. (2025) [44] and Joseph (2025) [45] document systematic gender and racial bias in clinical AI and machine learning algorithms, while the landmark study by Obermeyer et al. (2019) [46] demonstrated that a widely deployed clinical algorithm systematically underestimated illness severity in Black patients due to biassed training targets. In the obesity domain, differential model performance across demographic subgroups has not been systematically reported in the literature reviewed. We therefore recommend that equity-stratified performance reporting become a mandatory publication standard for AI obesity tools, alongside diverse training cohort requirements and community engagement in deployment design. Health equity must be a design criterion, not an afterthought.

**Figure 5:**
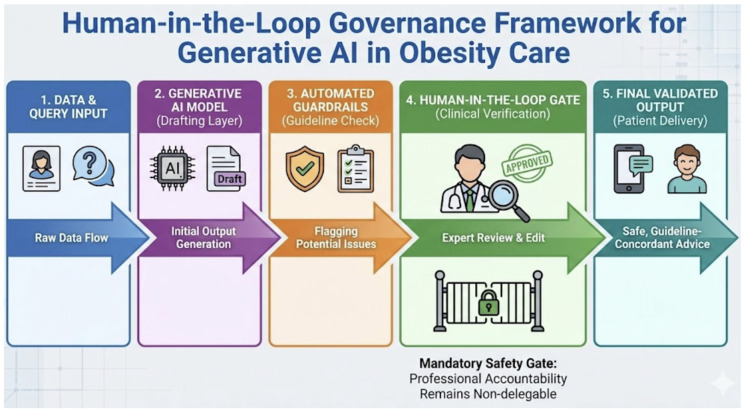
Human-in-the-loop (HITL) governance framework for generative AI in obesity care. The five-stage pipeline proceeds from (1) raw data and query input through (2) generative AI model output (drafting layer), (3) automated guardrails performing guideline concordance checks, (4) the mandatory human-in-the-loop safety gate at which clinicians review, edit, and approve AI-generated content, to (5) final validated patient-facing output. Professional accountability is non-delegable at Stage 4, ensuring that AI functions as a clinical co-pilot rather than an autonomous decision-maker. This architecture is proposed as a minimum governance standard for patient-facing AI applications in obesity management, consistent with WHO ethical AI principles and regulatory expectations for digital therapeutics. Abbreviations: HITL—human-in-the-loop; AI—artificial intelligence; LLM—large language model; WHO—World Health Organization.

The regulatory landscape for AI-based obesity digital therapeutics in the United States is evolving rapidly. The FDA’s Software as a Medical Device (SaMD) framework, supported by the Digital Health Center of Excellence, provides the primary regulatory pathway for AI/ML-based obesity tools. The 2021 AI/ML-Based SaMD Action Plan [15] introduced the concept of Pre-Determined Change Control Plans (PCCPs), enabling adaptive algorithms to update within pre-approved parameters without requiring a new regulatory submission—a critical enabler for AIBC platforms that learn from user data. The 2025 clearance of Signos [40] establishes a concrete regulatory precedent. However, post-market surveillance requirements for cleared AI obesity DTx remain less stringent than for Class II medical devices, and no EU-wide regulatory framework specifically addresses AI-based obesity DTx at the time of writing. We recommend that regulatory bodies prioritise expedited review pathways for equity-focused AI obesity DTx and establish post-market performance surveillance as a mandatory clearance condition. To mitigate the risk of generative hallucinations, the ‘Human-in-the-loop’ (HITL) architecture must be operationalized as a mandatory safety gate [14]. In this framework, LLM-generated patient education materials are not delivered directly; instead, they serve as ‘drafting layers’ for multidisciplinary teams. Professional accountability remains non-delegable; thus, AI tools should be viewed as ‘clinical co-pilots’ that synthesise data while the final therapeutic signature remains human-verified [49], as operationalised in the HITL framework illustrated in Figure 5.

This review is limited by its narrative design, which does not provide exhaustive retrieval or formal risk-of-bias assessment across all included studies. The heterogeneity of populations, data modalities, model architectures, and outcome definitions limits comparability and precludes quantitative synthesis. In addition, publication bias and selective reporting of model performance may inflate apparent effectiveness, particularly in early-stage digital interventions. A further limitation is the rapid pace of the field: several studies cited here were published as preprints or conference abstracts and have not yet undergone full peer review, which should be considered when interpreting reported effect sizes. Additionally, the predominance of studies from high-income settings—particularly the United States, South Korea, and the United Kingdom—limits the generalisability of conclusions to low- and middle-income countries where obesity prevalence is rising most rapidly and health system infrastructure for AI deployment differs fundamentally. The review did not formally assess the reporting quality of included AI studies against frameworks such as TRIPOD or CONSORT-AI, which would have strengthened the appraisal of model validation claims. Future systematic reviews incorporating formal quality assessment and meta-analysis will be essential to consolidate the evidence base as it matures.

## 5. Conclusions

Across the obesity care continuum, AI demonstrates meaningful but uneven translational promise. The most robust signals to date have emerged in EHR-based risk prediction and selected digital lifestyle interventions, where evidence includes large retrospective cohorts and at least one RCT. Multimodal phenotyping and pathway-level orchestration remain scientifically compelling but have not yet been validated in prospective, phenotype-guided treatment allocation trials. Routine clinical adoption across all four domains will require external validation on local populations, equity-stratified performance reporting, explicit bias auditing, and human-in-the-loop oversight for patient-facing generative systems. Future research should prioritise long-term effectiveness, cost-effectiveness, and implementation studies that measure equity and safety outcomes alongside weight and metabolic endpoints. When embedded within appropriate governance frameworks and multidisciplinary care models, AI holds the potential to support genuinely personalised obesity treatment pathways—but this potential must be realised through rigorous validation rather than assumed from proof-of-concept performance.

## Data Availability

No new data were created or analysed in this study. Data sharing is not applicable to this article.

## Supplementary Materials

The following supporting information can be downloaded at: https://www.mdpi.com/article/10.3390/diagnostics16101461/s1, Table S1: Summary of key studies (2024–2026) across the four AI domains in obesity care. Studies are organised by domain and ranked by translational readiness; Supplementary Materials: Translational readiness categories; Abbreviations; Domain colour coding.

## Abbreviations

The following abbreviations are used in this manuscript:

AI	artificial intelligence
AIBC	AI-enabled behavioural coaching
AUC	area under the receiver operating characteristic curve
BMI	body mass index
CDSS	clinical decision support system
CGM	continuous glucose monitoring
CNN	convolutional neural network
CT	computed tomography
DPP	Diabetes Prevention Programme
DTx	digital therapeutics
DXA	dual-energy X-ray absorptiometry
EHR	electronic health record
FDA	US Food and Drug Administration
GLP-1 RA	glucagon-like peptide-1 receptor agonist
HITL	human-in-the-loop
JITAI	just-in-time adaptive intervention
KNHANES	Korea National Health and Nutrition Examination Survey
LMIC	low- and middle-income countries
LLM	large language model
MBS	metabolic and bariatric surgery
ML	machine learning
DL	deep learning
MRI	magnetic resonance imaging
NHANES	National Health and Nutrition Examination Survey
PRS	polygenic risk score
RCT	randomised controlled trial
SES	socioeconomic status
SHAP	SHapley Additive exPlanations
SNP	single nucleotide polymorphism
WHO	World Health Organization
XGBoost	extreme gradient boosting

## References

[1] NCD Risk Factor Collaboration (NCD-RisC) (2024). Worldwide trends in underweight and obesity from 1990 to 2022: A pooled analysis of 3663 population-representative studies with 222 million children, adolescents, and adults. Lancet.

[2] Topol E.J. (2019). High-performance medicine: The convergence of human and artificial intelligence. Nat. Med..

[3] Rajpurkar P., Chen E., Banerjee O., Topol E.J. (2022). AI in health and medicine. Nat. Med..

[4] Obermeyer Z., Emanuel E.J. (2016). Predicting the Future—Big Data, Machine Learning, and Clinical Medicine. N. Engl. J. Med..

[5] Wójcik S., Rulkiewicz A., Pruszczyk P., Lisik W., Poboży M., Domienik-Karłowicz J. (2023). Beyond ChatGPT: What does GPT-4 add to healthcare? The dawn of a new era. Cardiol. J..

[6] Hallock R.K., Griebeler M.L., Burguera B., Cabandugama P. (2026). Transforming obesity medicine with artificial intelligence: Personalization, precision and the path ahead. Obes. Endocrinol..

[7] Jia X., Lin H., Ding Y., Hu C., Wang S., Li M., Xu Y., Xu M., Huang F., Shen F. (2025). Phenotyping obesity through a two-dimensional tree structure reveals cardiometabolic heterogeneity. Cell Rep. Med..

[8] Kim J.H., Lee H., Hwang J., Yon D.K., Rhee S.Y. (2025). Multimodal and Multidimensional Artificial Intelligence Technology in Obesity. J. Obes. Metab. Syndr..

[9] Shortliffe E.H., Sepúlveda M.J. (2018). Clinical Decision Support in the Era of Artificial Intelligence. JAMA.

[10] Akbarialiabad H., Pasdar A., Murrell D.F., Mostafavi M., Shakil F., Safaee E., Leachman S.A., Haghighi A., Tarbox M., Bunick C.G. (2025). Enhancing randomized clinical trials with digital twins. npj Syst. Biol. Appl..

[11] Kotov A., Idalski Carcone A., Towner E. (2024). Neural Conversational Agent for Weight Loss Counseling: Protocol for an Implementation and Feasibility Study. JMIR Res. Protoc..

[12] Nahum-Shani I., Smith S.N., Spring B.J., Collins L.M., Witkiewitz K., Tewari A., Murphy S.A. (2018). Just-in-Time Adaptive Interventions (JITAIs) in Mobile Health: Key Components and Design Principles for Ongoing Health Behavior Support. Ann. Behav. Med..

[13] Wójcik S., Rulkiewicz A., Pruszczyk P., Lisik W., Poboży M., Domienik-Karłowicz J. (2024). Reshaping medical education: Performance of ChatGPT on a PES medical examination. Cardiol. J..

[14] Crigger E., Reinbold K., Hanson C., Kao A., Blake K., Irons M. (2022). Trustworthy Augmented Intelligence in Health Care. J. Med. Syst..

[15] U.S. Food and Drug Administration (2021). Artificial Intelligence/Machine Learning (AI/ML)-Based Software as a Medical Device (SaMD) Action Plan.

[16] World Health Organization (2025). WHO Guideline on the Use of GLP-1 Receptor Agonists for the Management of Obesity.

[17] Azmi S., Kunnathodi F., Alotaibi H.F., Alhazzani W., Mustafa M., Ahmad I., Anvarbatcha R., Lytras M.D., Arafat A.A. (2025). Harnessing Artificial Intelligence in Obesity Research and Management: A Comprehensive Review. Diagnostics.

[18] Huang L., Huhulea E.N., Abraham E., Bienenstock R., Aifuwa E., Hirani R., Schulhof A., Tiwari R.K., Etienne M. (2025). The Role of Artificial Intelligence in Obesity Risk Prediction and Management: Approaches, Insights, and Recommendations. Medicina.

[19] Gupta M., Phan T.-L.T., Bunnell H.T., Beheshti R. (2022). Obesity Prediction with EHR Data: A deep learning approach with interpretable elements. ACM Trans. Comput. Healthc..

[20] Niakan Kalhori S.R., Najafi F., Hasannejadasl H., Heydari S. (2025). Artificial intelligence-enabled obesity prediction: A systematic review of cohort data analysis. Int. J. Med. Inform..

[21] Jeong S., Choi Y.-J. (2024). Investigating the Influence of Heavy Metals and Environmental Factors on Metabolic Syndrome Risk Based on Nutrient Intake: Machine Learning Analysis of Data from the Eighth Korea National Health and Nutrition Examination Survey (KNHANES). Nutrients.

[22] Jeon J., Lee S., Oh C. (2023). Age-specific risk factors for the prediction of obesity using a machine learning approach. Front. Public Health.

[23] Netayawijit P., Chansanam W., Sorn-In K. (2025). Interpretable Machine Learning Framework for Diabetes Prediction: Integrating SMOTE Balancing with SHAP Explainability for Clinical Decision Support. Healthcare.

[24] Hall K.D., Farooqi I.S., Friedman J.M., Klein S., Loos R.J.F., Mangelsdorf D.J., O’Rahilly S., Ravussin E., Redman L.M., Ryan D.H. (2022). The energy balance model of obesity: Beyond calories in, calories out. Am. J. Clin. Nutr..

[25] Cauchi D., Glonti K., Petticrew M., Knai C. (2016). Environmental components of childhood obesity prevention interventions: An overview of systematic reviews. Obes. Rev..

[26] Chen Z., Zhang T., Dazard J.-E., Ponnana S.R., Dong W., Moorthy S., Sirasapalli S.K., Khraishah H., Deo S., Rajagopalan S. (2025). AI-Enhanced Analysis of Built Environment Imagery and Neighborhood Obesity in US Cities. JAMA Netw. Open.

[27] Dahu B.M., Khan S., Toubal I.E., Alshehri M., Martinez-Villar C.I., Ogundele O.B., Sheets L.R., Scott G.J. (2024). Geospatial Modeling of Deep Neural Visual Features for Predicting Obesity Prevalence in Missouri: Quantitative Study. JMIR AI.

[28] Ghorbany S., Hu M., Yao S., Sisk M., Wang C., Zhang K., Nguyen Q.C. (2025). Data driven assessment of built environment impacts on urban health across United States cities. Sci. Rep..

[29] Vahid F., Loyola-Leyva A., Tur J., Bouzas C., Devaux Y., Malisoux L., Garcia S., De Carvalho M., Ródenas-Munar M., Turner J. (2026). Multimodal (Bio)Markers and Risk of Obesity—A Comprehensive Scoping Review. Adv. Nutr..

[30] Ruan X., Lu S., Wang L., Wen A., Murali S., Liu H. (2025). Deep Phenotyping of Obesity: Electronic Health Record–Based Temporal Modeling Study. J. Med. Internet Res..

[31] Xiao L., Liang S., Zeng L., Cai S., Wang J., Hong C., Li Y., Li R., Jiang P., Xie Z. (2026). A machine learning-derived polygenic risk score reveals that healthy lifestyle counteracts obesity-related mortality. npj Digit. Med..

[32] Wang M.H. (2026). Artificial Intelligence Across the Obesity Continuum: From Mechanistic Insights to Global Precision Prevention and Therapy. Obesity.

[33] Chew H.S.J., Chew N.W., Loong S.S.E., Lim S.L., Tam W.S.W., Chin Y.H., Chao A.M., Dimitriadis G.K., Gao Y., So J.B.Y. (2024). Effectiveness of an Artificial Intelligence-Assisted App for Improving Eating Behaviors: Mixed Methods Evaluation. J. Med. Internet Res..

[34] Noh E., Won J., Jo S., Hahm D.-H., Lee H. (2023). Conversational Agents for Body Weight Management: Systematic Review. J. Med. Internet Res..

[35] Pujia C., Ferro Y., Mazza E., Maurotti S., Montalcini T., Pujia A. (2025). The Role of Mobile Apps in Obesity Management: Systematic Review and Meta-Analysis. J. Med. Internet Res..

[36] Colaone E., Belluzzi E., Pozzuoli A., Roma E., Bortolato E., Biz C., Ruggieri P. (2025). Effectiveness of Telemedicine Nursing Interventions in the Management of Overweight and Obesity in Adults: A Systematic Review and Meta-Analysis. Curr. Obes. Rep..

[37] Mathioudakis N., Lalani B., Abusamaan M.S., Alderfer M., Alver D., Dobs A., Kane B., McGready J., Riekert K., Ringham B. (2025). An AI-Powered Lifestyle Intervention vs Human Coaching in the Diabetes Prevention Programme: A Randomized Clinical Trial. JAMA.

[38] Obesity Medicine Association (2025). Obesity Algorithm 2025.

[39] Nadolsky K., Garvey W.T., Agarwal M., Bonnecaze A., Burguera B., DeGeeter Chaplin M., Griebeler M.L., Harris S.R., Schellinger J.N., Simonetti J. (2025). American Association of Clinical Endocrinology Consensus Statement: Algorithm for the Evaluation and Treatment of Adults with Obesity/Adiposity-Based Chronic Disease–2025 Update. Endocr. Pract..

[40] US Food and Drug Administration (2025). FDA Clears Signos AI-Driven Glucose Monitoring for Weight Management [News Release]. https://www.cnbc.com/2025/08/20/fda-approves-signos-glucose-monitoring-for-weight-loss.html.

[41] Allurion Technologies (2025). AllurionMeds: AI-Driven Coaching with GLP-1 Prescribing [Press Release]. https://investors.allurion.com/news/news-details/2025/Allurion-Announces-Plans-To-Combine-GLP-1-Therapy-With-The-Allurion-Balloon-to-Treat-Obesity/default.aspx.

[42] IQVIA Institute for Human Data Science (2025). Obesity and Weight Management 2025 Report.

[43] Khera R., Butte A.J., Berkwits M., Hswen Y., Flanagin A., Park H., Curfman G., Bibbins-Domingo K. (2023). AI in Medicine—*JAMA*’s Focus on Clinical Outcomes, Patient-Centered Care, Quality, and Equity. JAMA.

[44] Otokiti A.U., Shih H.-J., Williams K.S. (2025). Gender and racial bias unveiled: Clinical artificial intelligence (AI) and machine learning (ML) algorithms are fanning the flames of inequity. Oxf. Open Digit. Health.

[45] Joseph J. (2025). Algorithmic bias in public health AI: A silent threat to equity in low-resource settings. Front. Public Health.

[46] Obermeyer Z., Powers B., Vogeli C., Mullainathan S. (2019). Dissecting racial bias in an algorithm used to manage the health of populations. Science.

[47] World Health Organization (2021). Ethics and Governance of Artificial Intelligence for Health: WHO Guidance.

[48] Wang X., Bai Y., Li W., Yang C., Zhang L., Zhu H., Bao R., Jiang Y., Wang F., Wang H. (2025). Effect of artificial intelligence driven therapeutic lifestyle changes (AI-TLC) intervention on health behavior and health among obesity pregnant women in China: A randomized controlled trial protocol. Front. Public Health.

[49] Huang L., Yu W., Ma W., Zhong W., Feng Z., Wang H., Chen Q., Peng W., Feng X., Qin B. (2025). A Survey on Hallucination in Large Language Models: Principles, Taxonomy, Challenges, and Open Questions. ACM Trans. Inf. Syst..

