# Supplementary material for: Artificial Intelligence-Based Risk Stratification in Obesity Care: From Diagnosis to Personalised Treatment Pathways
Source: Diagnostics (Basel). 2026 May 11;16(10):1461. doi: 10.3390/diagnostics16101461 (PMC13206304; doi:10.3390/diagnostics16101461)
Supplement: Supplementary file 1 [file diagnostics-16-01461-s001.zip › diagnostics-4214470-supplementary.pdf]

Table S1. *Summary of key studies (2024–2026) across the four AI domains in obesity care. Studies are organised by domain and ranked by translational readiness.*

Domain	Study (First Author, Year)	Study design	Population / Setting	Sample size (n)	Primary data modality	Key AI Method	Primary outcome	Explainability method	External validation	Prospective validation	Translational readiness
1 – EHR Prediction	Kalhori et al., 2025	Systematic review (10 studies)	Mixed (paediatric + adult cohorts)	411,000	EHR (structured)	Multiple ML methods	ML models forecast obesity 1–5 yrs ahead	Variable across studies	Limited (some studies)	No	Exploratory / Clinical validation
1 – EHR Prediction	Choong et al., 2024	Retrospective claims-EMR linkage	Adults, US (insurance claims)	692,119	Claims data (structured)	LR, LASSO, XGBoost, RF	Obesity status prediction from claims data	None reported	No	No	Exploratory
1 – EHR Prediction	Netayawijit et al., 2025	Retrospective cohort (NHANES)	Adults, US (NHANES)	NHANES sample	EHR + behavioural variables	ML ensemble + SMOTE	Interpretable obesity/ diabetes risk factors	SHAP + LIME	No	No	Exploratory
2 – Built Environment	Chen et al., 2025	Cross-sectional geospatial	Census tracts, 10 US metros	>1,000 census tracts	Satellite + Street View imagery	Deep CNN architectures	20–30% explained variance in obesity prevalence	None reported	Partial (cross-city transfer)	No	Exploratory
2 – Built Environment	Dahu et al., 2024	Cross-sectional geospatial	Census tracts, Missouri, US	1,052 census tracts	Satellite imagery (Sentinel-2, 10 m)	ResNet-50 + spatial econometrics	R^2 +15–25% over non-spatial models	None reported	No (internal spatial CV)	No	Exploratory
2 – Built Environment	Ghorbany et al., 2025	Cross-sectional geospatial	19 US urban areas (diverse climates)	19 cities	Street View + Landsat satellite	Multi-modal CNN ensemble	~25% variance; ≥30% green coverage protective	None reported	Partial (multi-city)	No	Exploratory
3 – Multimodal Phenotyping	Xiao et al., 2026	Retrospective cohort (UK Biobank)	European ancestry, UK Biobank	482,700	Genomics + phenotypic + lifestyle	ML-derived PRS	AUC +12–18% over standard PRS	None reported	No (internal split)	No	Exploratory
3 – Multimodal Phenotyping	Vahid et al., 2026	Scoping review (>100 studies)	Multiple cohorts (global)	Multiple cohorts	Genomics + metabolomics + imaging	Regularised regression, tree ensembles, NN	AUC gain 0.08–0.15 multimodal vs unimodal	Variable	Variable across studies	No	Exploratory / Clinical validation
3 – Multimodal Phenotyping	Jia et al., 2025	Retrospective cohort	Adults, multi-site (China)	18,733	Routine clinical data (EHR)	DDRTree unsupervised clustering	5 metabolic phenotypes with distinct CVD/T2D risk	None reported	No (internal)	No	Exploratory
3 – Multimodal Phenotyping	Hosseini et al., 2025	Retrospective EHR cohort	Adults, EHR-based	Large EHR cohort	EHR (structured: dx, meds, anthro)	EHR-based deep phenotyping	Multidimensional obesity subtypes without omics	None reported	No (internal)	No	Exploratory
3 – Multimodal Phenotyping	Kim et al., 2025	Comprehensive review	Multiple populations (global)	Multiple studies	Multi-modal (imaging, omics, sensors, text)	Multiple multimodal AI frameworks	Multimodal > unimodal in 91% of studies (6–33% gain)	Variable	Variable	No	Exploratory
4 – AIBC / DTx	Mathioudakis et al., 2025	RCT (AI vs human DPP)	Adults with prediabetes + overweight/obesity, US	368	Wearables + self-report + app	AI-powered adaptive coaching	Non-inferior weight loss (≥5%) and HbA1c	None reported	N/A (single-site RCT)	Yes (prospective RCT)	Clinical validation (RCT)
4 – AIBC / DTx	Chew et al., 2024	Mixed-methods evaluation	Adults, app users	App user cohort	App-based dietary self-report	AI-assisted dietary app	Improved eating behaviours	None reported	No	Partial (prospective app use)	Exploratory / Feasibility
4 – AIBC / DTx	Pujia et al., 2025	Systematic review + meta-analysis	Adults with overweight/ obesity (multiple RCTs)	Multiple RCTs pooled	Smartphone app interventions	Mobile app-based interventions	Modest weight loss; hybrid > app-only	N/A (meta-analysis)	N/A (meta-analysis)	Yes (pooled RCTs)	Clinical validation
4 – AIBC / DTx	Wang X et al., 2025	RCT protocol	Pregnant women with obesity, China	Planned (protocol)	AI-TLC algorithmic coaching	AI-driven therapeutic lifestyle change	Health behaviour and gestational outcomes	N/A (protocol)	N/A (protocol)	Yes (planned RCT)	Protocol stage
Cross-cutting – Regulatory	FDA / Signos, 2025	Regulatory clearance	Adults, US (commercial)	N/A	CGM + app (real-time glucose)	AI + CGM for weight management	First FDA-cleared AI obesity DTx	N/A	N/A	N/A	Regulatory clearance

Translational readiness categories: *Exploratory = model development and internal validation only; Clinical validation = external validation, systematic review-level evidence, or RCT; Regulatory clearance = formal regulatory approval; Protocol stage = study design published, results pending.*

Abbreviations: *AIBC—AI-enabled behavioral coaching; AUC—area under the receiver operating characteristic curve; CGM—continuous glucose monitoring; CNN—convolutional neural network; CV—cross-validation; CVD—cardiovascular disease; DTx—digital therapeutics; DPP—Diabetes Prevention Programme; EHR—electronic health record; FDA—US Food and Drug Administration; LR—logistic regression; LSTM—long short-term memory; ML—machine learning; NN—neural network; PRS—polygenic risk score; RCT—randomized controlled trial; RF—random forest; SHAP—SHapley Additive exPlanations; T2D—type 2 diabetes.*

Domain colour coding: *Blue = Domain 1 (EHR Prediction); Green = Domain 2 (Built Environment); Orange = Domain 3 (Multimodal Phenotyping); Purple = Domain 4 (AIBC / Digital Therapeutics); Grey = Cross-cutting (Regulatory).*
